# Multifunctional polyimide performance prediction based on explainable machine learning

**DOI:** 10.1002/smo2.70020

**Published:** 2025-10-30

**Authors:** Suisui Wang, Tianyong Zhang, Han Zhang, Wenxuan Zhu, Zixu Gu, Xufeng Huang, Hande Zhang, Bin Li, Jianhua Zhang

**Affiliations:** ^1^ School of Chemical Engineering and Technology Tianjin University Tianjin China; ^2^ School of Microelectronics Shanghai University Shanghai China

**Keywords:** cut‐off wavelength, machine learning, performance prediction, polyimides, thermodynamics

## Abstract

Polyimides (PIs) are widely used in the microelectronics field due to their excellent comprehensive performance and the diversity and designability of their structures. In flexible substrate applications, designing the molecular structure to balance thermodynamic and optical properties is the most critical part of the PI design process. To accelerate the discovery of high‐performance PIs, we established predictive models for glass transition temperature (T_g_), cut‐off wavelength (CW), and coefficient of thermal expansion (CTE) using various machine learning algorithms. The optimal predictive models for the three properties demonstrated high accuracy and stability in both test set predictions and cross‐validation results. Additionally, the interpretability of the three optimal models was analyzed using the SHAP method, and the accuracy and generalization ability of the models were validated using several novel PIs. By combining the three models, predictions were made for multiple PIs, leading to the selection and synthesis of PIs with excellent comprehensive performance. 135 novel PIs were designed and their key properties were obtained without the need for experimental verification. The predictive models established in this study can assist researchers in quickly determining the T_g_, CW and CTE of PIs, thereby facilitating the swift identification of promising candidates for further development.

## Graphical abstract

Utilizing interpretable machine learning algorithms to develop predictive models for the glass transition temperature, cut‐off wavelength, and coefficient of thermal expansion of polyimides can assist in the design of novel high‐performance polyimides. This approach facilitates the swift identification of promising candidates for further development and provides new insights into the discovery of high‐performance polymers for the future.

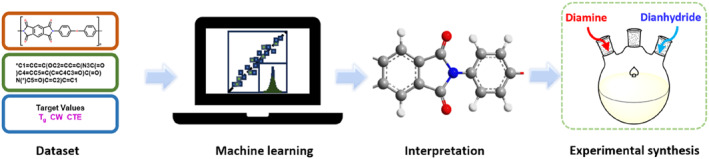

## 1 INTRODUCTION

The rapid development of flexible display technology has promoted innovations in flexible substrate materials. As an essential component of flexible display devices, flexible substrates impose stringent requirements on the thermomechanical, optical, and other properties of the materials.[^1^, ^2^, ^3^] As a core material for flexible electronic devices, polyimide (PI) must meet a variety of performance requirements. Flexible display substrates require high light transmittance (e.g., >80% T@430 nm) and near‐colorless characteristics to match the optical demands of display technologies such as OLEDs. Moreover, during the manufacturing process of flexible display devices, which involves high‐temperature processes (e.g., thin‐film transistor deposition), the glass transition temperature (T_g_) of the PI substrate must exceed 300°C. Additionally, the coefficient of thermal expansion (CTE) of PI should be matched with that of metal electrodes to prevent interlayer delamination or warpage caused by thermal stress. Therefore, PI with high transparency, high T_g_, and low CTE is considered to be one of the most promising transparent substrate materials for flexible displays.[^4^, ^5^, ^6^, ^7^, ^8^, ^9^] The charge transfer interactions within and between the PI main chain molecules can lead to poor transparency of PI films in the visible light region.[^10^, ^11^, ^12^] To overcome this issue of deeper color, substituents with high electronegativity (such as trifluoromethyl or sulfonyl groups)[^13^, ^14^, ^15^] or aliphatic units (such as cyclohexyl groups)[^16^, ^17^, ^18^, ^19^, ^20^] can be introduced into the PI molecular chain to reduce or even eliminate charge transfer interactions. However, reducing charge transfer interactions can also decrease the compactness of the molecular chains, thus diminishing the thermal resistance of PIs or significantly increasing the CTE.[^21^, ^22^, ^23^] Designing molecular structures to balance thermomechanical and optical properties is the most critical aspect of PI design. Traditional methods require extensive experimental exploration and screening of acceptable structures, which can be time‐consuming and costly. Simulation methods such as density functional theory (DFT) and molecular dynamics (MD) can alleviate this problem to some extent, but these methods still suffer from drawbacks such as being time‐consuming and highly dependent on expert experience for the selection of simulation parameters.[^24^, ^25^, ^26^, ^27^]

In recent years, with the rapid development of machine learning (ML), using ML algorithms to assist in the structural design and screening of materials can accelerate the development of new materials.[^28^, ^29^, ^30^, ^31^] Therefore, researchers have attempted to use computational methods to effectively predict the properties of novel PIs. By leveraging data‐driven discovery to find PI with superior comprehensive performance before laboratory synthesis and testing, the research and development cycle can be shortened, shifting from traditional trial and error experiments to theory guided experimentation.[^32^, ^33^] Most current work focuses on predicting the glass transition temperature (T_g_), as PIs are among the most heat‐resistant polymers. Researchers use ML techniques to establish predictive models for T_g_. For example, Liu^[34]^ collected 54 types of aromatic heterocyclic PIs to develop a quantitative structure–property relationship (QSPR) model and used the backpropagation algorithm of artificial neural networks to predict T_g_, achieving a root mean square error (RMSE) of 16.4°C and a correlation coefficient (R) of 0.937 on the test set of 18 samples. Wen et al.^[35]^ collected 225 types of PI from the literature, obtained their SMILES (Simplified Molecular Input Line Entry System) representations for monomers, and generated 1342 molecular descriptors as feature inputs for the ML model. Their LASSO (Least Absolute Shrinkage and Selection Operator) model combined with the bagging method resulted in an average error of 18°C in T_g_ prediction, indicating that the ML model has good predictive capabilities. However, concerns over the generalization ability of the obtained model arise due to the small dataset used, and there is a strong desire to further improve the model using a larger dataset.[^36^, ^37^]

It can be seen from the above discussion that all previous studies have encountered various issues when evaluating a single property (T_g_) of PI. Moreover, few efforts have been dedicated to exploring other properties simultaneously. Zhang et al.^[38]^ used 7 ML algorithms to predict T_g_ and cut‐off wavelength (CW) (652 data points on T_g_ and 201 data points on CW). The optimal model had an RMSE of 33.92°C for the predicted T_g_ and 17.18 nm for the CW. Tao et al.^[39]^ employed computational approaches combining ML and MD simulations to establish multiple ML models for experimentally reported thermal and mechanical property values of PI, including T_g_, Young's modulus, and tensile yield strength. However, critical properties such as CW and CTE were excluded from the modeling framework. Notably, the RMSE for T_g_ predictions reached 35.2°C, indicating limitations in model accuracy for this key thermal property. Furthermore, when it comes to multiple properties, the evaluation of PI becomes even more laborious.

**FIGURE 1:**
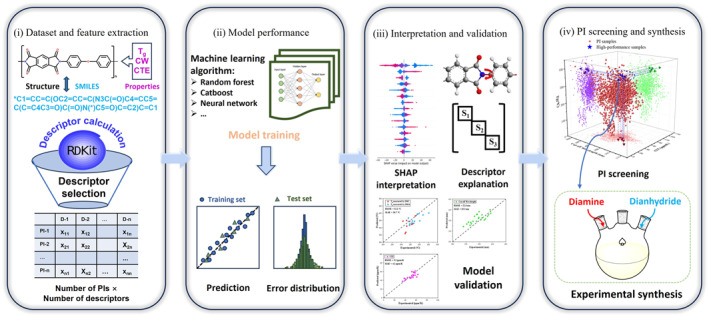
Workflow diagram for predicting the glass transition temperature, cut‐off wavelength and coefficient of thermal expansion of polyimide based on machine learning algorithms.

Overall, the prediction of PI properties remains challenged by limitations such as small dataset sizes, insufficient accuracy and generalization capabilities, and the absence of predictive models for critical properties such as CTE. We propose that expanding dataset scales and employing multiple ML methodologies can enhance model accuracy and generalizability. Furthermore, integrating multiple predictive models facilitates the screening of novel PIs with exceptional multifunctional performance.^[40]^ Faced with these difficulties and challenges, we recognize that screening for novel PIs with excellent multifunctional properties requires analysis through the integration of multiple predictive models. Thus, this paper collects a large amount of PI data (more than 1600) and uses various ML algorithms, including ensemble learning and neural networks to construct predictive models for T_g_, CW and CTE separately. Additionally, with the help of SHAP (SHapley Additive exPlanations),^[41]^ the importance of descriptors is extracted to explain the models, and the accuracy and practical applicability of the models are further evaluated using PI outside the dataset. Then, we screen PIs by integrating the three predictive models, synthesize PIs with superior overall performance in experiments, and measure their properties to validate the predictions of the ML models. Finally, 135 novel PIs were designed and their key properties were obtained without the need of experimental verification. Through the screening process, candidates with superior overall performance and synthetic accessibility, as well as single high‐performance PIs, were identified. This work predicts multiple key properties of PIs and demonstrates high accuracy and generalization ability in the models, which can be used to discover new PI materials with ideal performance. The workflow diagram is shown in Figure 1.

## 2 RESULTS AND DISCUSSION

### 2.1 Dataset and feature extraction

**FIGURE 2:**
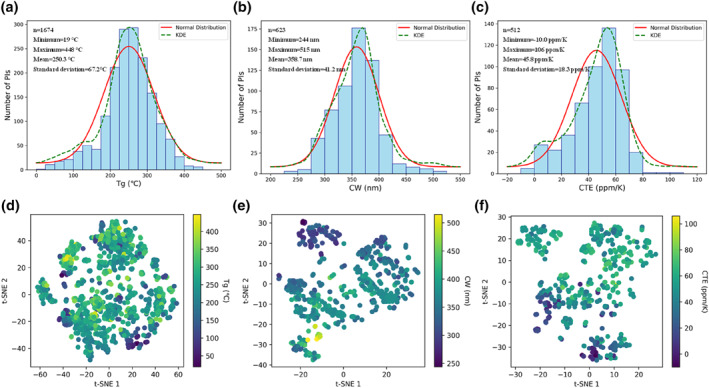
Distribution of the T_g_, CW, and CTE datasets and visualization of the chemical space, where n is the sample size of the data. 2D visualization based on molecular descriptors using the t‐SNE algorithm. (a) and (d) T_g_ dataset; (b) and (e) CW dataset; (c) and (f) CTE dataset. CTE, coefficient of thermal expansion; CW, cut‐off wavelength; T_g_, glass transition temperature.

In this paper, we have collected a total of 1674 T_g_ data points, 623 CW data points, and 512 CTE data points. The detailed information of their SMILES and data sources can be found on the website (https://github.com/wsstju/SMILES‐and‐descriptor). The data distributions of the three properties are shown in Figure 2a–c. The collected data span a wide range, with T_g_ values ranging from 19 to 448°C, CW values from 244 to 515 nm, and CTE values from −10 to 106 ppm/K. The kernel density estimation (KDE) curves of these three properties are close to normal distribution curves, with most data clustered around the mean. The amount of data decreases as the values move away from the mean. Most CTE values are less than 70 ppm/K, but there are still data points with relatively higher CTE values, contributing to the diversity of the dataset. The chemical space distribution of these three properties is shown in Figure 2d–f, where the color of the data points changes from purple to yellow as the values of T_g_, CW, and CTE increase. Data points with similar values tend to cluster together in the chemical space, indicating structural similarity. The T_g_ dataset is the largest and occupies a more extensive and diverse region within the chemical space, reflecting a wider variety of structures. Although the CW and CTE datasets are smaller than the T_g_ dataset, each still contains over 500 data points. They essentially cover all possible structures from the literature of the past 2 decades, having a limited impact on the model's generalization. As shown in the t‐SNE plot of Morgan molecular fingerprints (Supporting Information S1: Figure S1), molecules that are closer in the spatial distribution exhibit higher structural similarity and correspondingly closer performance data.^[42]^ The distribution in the chemical space also demonstrates the structural diversity of PI repeating units, which includes many significantly distinct structures, such as aliphatic PIs and aromatic PIs. These structures are derived from approximately 400 different articles, combining various projects and showcasing remarkable diversity.

A total of 210 molecular descriptors were calculated using RDKit, with detailed information about the descriptors available at https://github.com/wsstju/SMILES‐and‐descriptor. After removing descriptors with high similarity and significant overlap of information based on the variance selection method, the T_g_ dataset retained 131 descriptors, the CW dataset retained 140 descriptors, and the CTE dataset retained 144 descriptors. For the ensemble learning algorithms, the most influential descriptors for the prediction results were identified and selected using the feature importance method, serving as inputs for the predictive models. The number of descriptors finally obtained varies across different algorithms, as detailed in Supporting Information S1: Table S1. Since neural networks can automatically learn and extract the most important descriptors, the descriptors selected after variance selection were directly used as inputs.

### 2.2 Model performance

The goodness of fit for each model was evaluated using the statistical parameters coefficient of determination (*R* ^2^), mean absolute error (MAE) and RMSE. Supporting Information S1: Figure S2 shows the accuracy of predicting T_g_ using different algorithms on both the training and test sets, as well as the error distribution. Generally, the closer the points are to the dashed line, the smaller the error between the predicted and experimental values, indicating higher model accuracy. Most of the predicted errors are concentrated within the range of 0–25°C, suggesting that the models predict accurately and possess good robustness.

**FIGURE 3:**
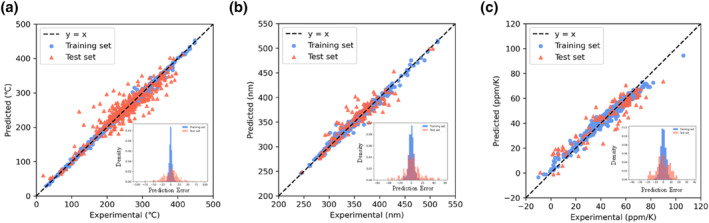
(a) Results of the glass transition temperature prediction models trained by CATB algorithm and prediction error distribution of training set and test set. (b) Results of the cut‐off wavelength prediction models trained by CATB algorithm and PE distribution of training set and test set. (c) Results of the coefficient of thermal expansion prediction models trained by the random forest algorithm and PE distribution of the training set and test set.

The performance of the T_g_ prediction models on the training and test sets, along with the cross‐validation results, are presented in Supporting Information S1: Table S2. Except for the Deep Neural Network (DNN) model, the RMSE for the test set and 10‐fold cross‐validation of the remaining ensemble learning models was within 30°C, further indicating that these models have good predictability and stability. Because random forest (RF) and ET are built‐in learners based on the Bagging algorithm, they reduce the correlation between base learners through bootstrap sampling; LGBM, XGB, and CATB are built‐in learners based on the Boosting algorithm, optimizing the performance of base learners iteratively through gradient boosting. These models combine multiple base learners to enhance model prediction performance. However, all these models exhibited overfitting, with test set and cross‐validation results significantly lower than the training results, because of the complexity introduced by the numerous base learners leading to overfitting. DNN, a neural network containing multiple hidden layers, can learn complex nonlinear relationships and patterns, but requires substantial data and computational resources for training. For simpler tasks, the DNN might be overly complex, leading to a drop in performance. The results indicate that the T_g_ prediction model trained using the CATB algorithm is more accurate and stable (Figure 3a), achieving an accuracy of *R* ^2^ = 0.865, MAE = 18.1°C, and RMSE = 25.6°C on the test set.

Supporting Information S1: Figure S3 shows the accuracy of predicting CW using different algorithms on both the training and test sets, as well as the error distribution. The majority of the predicted errors are concentrated within the range of 0–20 nm. The performance of the CW prediction models on the training and test sets, along with the cross‐validation results, are presented in Supporting Information S1: Table S3. All models achieved an RMSE of less than 20 nm on the test set and in 10‐fold cross‐validation, further indicating that these models have good predictability and stability. Compared to other models, the model trained using the CATB algorithm exhibits higher accuracy and better stability (Figure 3b). The accuracy measured on the test set is *R* ^2^ = 0.874, MAE = 10.6 nm and RMSE = 15.0 nm. Supporting Information S1: Figure S4 shows the accuracy of predicting CTE using different algorithms on both the training and test sets, as well as the error distribution. The majority of the predicted errors are concentrated within the range of 0–10 ppm/K. The performance of the CTE prediction models on the training and test sets, along with the cross‐validation results, are presented in Supporting Information S1: Table S4. All models achieved an RMSE of less than 10 ppm/K on the test set and in 10‐fold cross‐validation, further indicating that these models have good predictability and stability. Compared to other models, the model trained using the RF algorithm exhibits higher accuracy and better stability (Figure 3c). The accuracy measured on the test set is *R* ^2^ = 0.855, MAE = 6.6 ppm/K and RMSE = 8.3 ppm/K.

We present the results of the comparison study in Table 1 (on test set). When comparing the two regression models, the evaluation metrics *R* ^2^, RMSE, and MAE each emphasizes distinct aspects. *R* ^2^ approaching 1 indicates a stronger explanatory power of the model for data variance. However, this does not necessarily imply that predicted values align more closely with true values. *R* ^2^ is sensitive to outliers and may be spuriously inflated by increased model complexity (e.g., overfitting). In contrast, RMSE and MAE quantify the absolute magnitude of prediction errors, directly reflecting predictive accuracy. These metrics are particularly suitable for scenarios requiring precise evaluation of PI property predictions. Consequently, our analysis prioritizes RMSE and MAE as primary evaluation criteria. As shown in Table 1, many existing models lack cross‐validation and rely on small datasets (<1000 samples). Strong performance on the test set alone cannot fully guarantee model reliability, as it may overlook sensitivity to data fluctuations. In contrast, our model demonstrated superior performance and robustness, validated through rigorous cross‐validation. Furthermore, we extend predictive capability to CTE, a critical property absent in prior studies.

**TABLE 1:** Comparison of PI performance predictions.

References/our work	T_g_ [°C]				CW [nm]				Cross‐validation^b^
	Samples^a^	*R* ^2^	RMSE	MAE	Samples	*R* ^2^	RMSE	MAE	
Qiu^[26]^	372	0.86		16					No
Wen^[35]^	225			25.5					No
Zhang^[38]^	652	0.711	33.92		201	0.620	17.18		Yes
Tao^[39]^	1870	0.78	35.2	23.8					Yes
Zhang^[40]^					281	0.920	21.7		No
Qiu^[43]^	687	0.95	28.04	19.34					Yes
Previous work^[44]^	1257	0.823	29.0	20.1					Yes
This work	1674	0.865	25.6	18.7	623	0.874	15.0	10.6	Yes

*Note*: Blank spaces denotes that the metrics were not covered.

Abbreviations: CW, cut‐off wavelength; MAE, mean absolute error; RMSE, root mean square error; T_g_, glass transition temperature.

^a^ The total data volume of the training set and test set.

^b^ Cross‐validation status: “Yes” indicates implementation with cross‐validation; “No” denotes absence of such validation.

### 2.3 Model interpretation and validation

**FIGURE 4:**
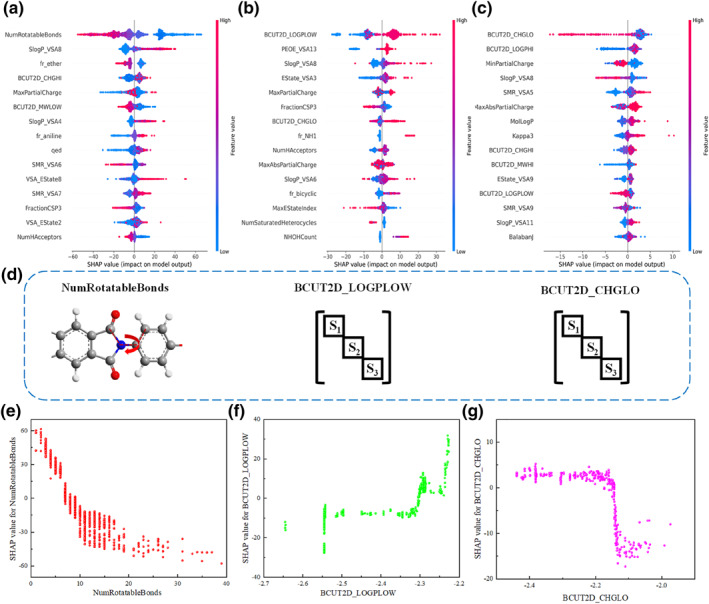
SHAP summary plots for the best prediction models and the relationship between the descriptors that have the most significant impact on the predicted values in the three models and their corresponding SHAP values: (a) Glass transition temperature prediction model, (b) cut‐off wavelength prediction model, (c) coefficient of thermal expansion prediction model, (d) Chemical interpretation of descriptors that have the most significant impact on predicted values, (e) SHAP value for NumRotatableBonds, (f) SHAP value for BCUT2D_LOGPLOW, (g) SHAP value for BCUT2D_CHGLO.

To further investigate the relationship between the microscopic structure of PI molecules and various properties, we used the SHAP method to perform interpretability analysis on the optimal models for T_g_, CW, and CTE, respectively, obtaining the importance ranking of descriptors for each model. The descriptors used in the three optimal models are shown in Supporting Information S1: Tables S5–S7. By selecting the top 15 most important descriptors, we took the average absolute value of the SHAP values for each feature as its importance, with the results shown in Supporting Information S1: Supporting Information S1: Figure S5–S7. However, based on feature importance alone, it is only possible to determine the degree of influence a descriptor has on the prediction result, not whether its specific numerical value exerts a positive or negative effect on the prediction value. Therefore, Summary Plots combining feature importance and feature impact were created using the SHAP method. These plots depict the SHAP value for each descriptor for every sample, focusing on the top 15 most important descriptors, as shown in Figure 4a–c. This allows for a better understanding of the overall pattern and enables the identification of predictive outliers. Each row represents a descriptor, and the *x*‐axis shows the SHAP values. Each dot represents a sample, with color indicating the feature value (red for high, blue for low). Figure 4d provides the chemical interpretation of the descriptors that have the most significant impact on the prediction values across the three models. Figure 4e–g shows the relationship between these three descriptors and their corresponding SHAP values.

From Figure 4a, it can be seen that the NumRotatableBonds descriptor is more important than other descriptors, having the most significant impact on T_g_, primarily related to the presence of single bonds in the molecule, usually carbon sp^3^–carbon sp^3^. The “free” rotation of atoms around single bonds allows the molecule to adopt different conformations, thus as the number of single bonds increases, flexibility also increases.^[45]^ From Figure 4b,c, it can be observed that the BCUT2D_LOGPLOW and BCUT2D_CHGLO descriptors are more important than other descriptors. BCUT2D_LOGPLOW incorporates the atomic pair logarithmic values of the partition coefficient (logP) into the Burden matrix and returns the lowest eigenvalue, while BCUT2D_CHGLO is the lowest eigenvalue of the Burden matrix weighted by atomic charge.^[46]^ S1, S2, and S3 represent substructures in the PI, which can encode atomic properties related to intermolecular interactions along their diagonals, including atomic charge, polarizability, H‐bond acceptor capacity, and H‐bond donor capacity.[^47^, ^48^] From Figure 4e–g, it can be seen that NumRotatableBonds exhibits a negative correlation with T_g_; as NumRotatableBonds increases, T_g_ continuously decreases. BCUT2D_LOGPLOW shows a positive correlation with CW. The value of BCUT2D_CHGLO around −2.17 indicates a transition in its influence on CTE, with SHAP values changing from positive to negative. However, there may also be some outliers, which could be influenced by other groups or spatial factors. Prediction values cannot focus solely on individual descriptors but are also influenced by other factors.

To verify the accuracy and generalization capability of the models, the three trained optimal models were used to predict the T_g_, CW and CTE of novel PI structures, with each property having at least 20 data points. A comparison of experimental values and prediction results is shown in Supporting Information S1: Figure S8, with detailed comparisons of experimental and predicted values available in Supporting Information S1: Table S8. The data points predicted by all three models are distributed near the dashed lines, indicating that the predicted values are close to the experimental values, suggesting good generalization capability of the models. Due to the fact that T_g_ values measured by dynamic mechanical analysis (DMA) are typically higher than those measured by differential scanning calorimetry (DSC), with differences ranging from a few degrees Celsius to tens of degrees, and since the model predicts T_g_ values obtained by DSC, the RMSE and MAE in Supporting Information S1: Figure S8a show a larger deviation relative to the cross‐validation results of the optimal model. In Supporting Information S1: Figure S8b, the RMSE and MAE for predicting CW are approximately 5 nm higher compared to the cross‐validation results of the optimal model. From Supporting Information S1: Figure S8c, it can be observed that the RMSE and MAE between the predicted and experimental CTE values are smaller, performing better than the cross‐validation results of the optimal model. Overall, these three models exhibit high accuracy and stability when predicting the properties of novel PIs.

### 2.4 High‐performance PI screening and synthesis

**FIGURE 5:**
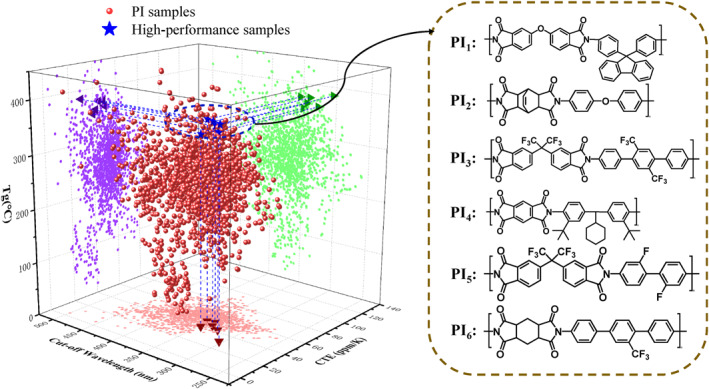
Three‐dimensional diagram of the three properties (glass transition temperature, cut‐off wavelength (CW) and coefficient of thermal expansion) of 1674 PIs and the screening of high‐performance PIs. On the right are six examples of PI structures with better comprehensive performance.

Applying the three trained ML models to 1674 types of PI yields their predicted T_g_, CW, and CTE values, allowing us to identify PIs that perform better in terms of multifunctionality. With three properties competing, the design space transforms into a three‐dimensional space, as shown in Figure 5. In this dataset, some PIs have only experimentally reported T_g_ values. However, in this 3D space, all three properties are necessary. Therefore, for CW and CTE, we supplement with the predicted values from the ML models. Using the complete data for T_g_, CW and CTE, all actual PIs are positioned accordingly in Figure 5. Although the final design space is a mix of experimental T_g_ values and predicted CW and CTE values, given the good predictive performance of the ML models, we consider it to be well‐structured and reliable. PIs with better comprehensive performance can be discovered from these, with some of the chemical structures displayed on the right side of Figure 5.

To verify the accuracy of the PI screening results, we prepared PI_1_ and PI_2_. Detailed experimental procedures can be found in the Supporting Information. The T_g_ of the PI was obtained using DSC with a heating rate of 10°C/min within the temperature range of 40–500°C; the optical transmission spectra of the films were measured using a UV‐visible spectrophotometer (GEN10S), with a scanning range of 200–800 nm; the CTE of the PI was measured using a Mettler Toledo‐TMA SDTA 2+ over a temperature range of 50–250°C and a heating rate of 5°C/min. The experimental data for T_g_, CW and CTE are shown in Supporting Information S1: Figure S9, with a comparison of the predicted and experimental values for PI_1_‐PI_6_ listed in Table 2. The PIs obtained through screening exhibit superior comprehensive performance, maintaining a lower CW and CTE while having a higher T_g_, and the discrepancy between the experimental values and the model's predictions is minimal. The results indicate that with the help of the model, it is possible to predict multiple unknown properties of PI and screen out high‐performance PIs with application potential.

**TABLE 2:** Comparison of experimental values and predicted values for T_g_, cut‐off wavelength (CW) and CTE of PI_1_‐PI_6_.

PI	T_g_ [°C]		CW [nm]		CTE [ppm/K]	
	Exp^a^	Pred^b^	Exp	Pred	Exp	Pred
PI_1_	351	370.3	379	354.4	43.4	48.7
PI_2_	377	378.0	304	304.7	22.2	30.0
PI_3_ ^[49]^	352	347.4	350	349.3	36.5	39.8
PI_4_ ^[16]^	374	372.0	338	339.8	47	45.0
PI_5_ ^[50]^	362.6	341.7	342	344.1	51.4	49.9
PI_6_ ^[49]^	359	354.0	332	327.2	39.8	42.2

Abbreviations: CTE, coefficient of thermal expansion; CW, cut‐off wavelength; PI, polyimide; T_g_, glass transition temperature.

^a^ Experimental value.

^b^ Predicted value.

To verify the findings that NumRotatableBonds, BCUT2D_LOGPLOW, and BCUT2D_CHGLO are more critical for PI performance, we analyzed the relationship between important descriptors and experimental values of PI_1_∼PI_6_ in Supporting Information S1: Figure S10. PIs with higher T_g_ values have been shown to have lower NumRotatableBonds values (i.e., PI_2_ and PI_4_), with none exceeding 5. PIs with larger cut‐off wavelengths also had larger values of BCUT2D_LOGPLOW (i.e., PI_1_). While individual descriptors do not solely determine the magnitude of T_g_, CW, or CTE, they must be considered in conjunction with other descriptors. However, the descriptors NumRotatableBonds, BCUT2D_LOGPLOW, and BCUT2D_CHGLO have the most significant impact on performance. Based on this insight, when designing the molecular structure of PI materials, it is advisable to carefully consider these three descriptors to enhance T_g_ values while reducing CW and CTE.

**FIGURE 6:**
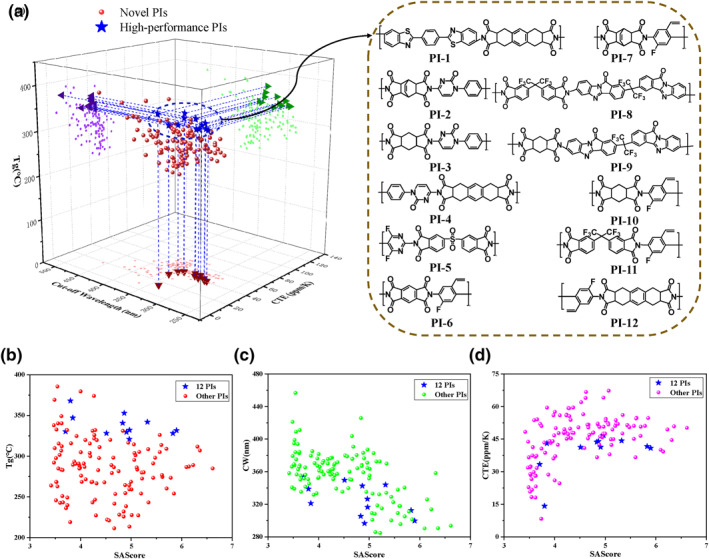
(a) Three‐dimensional (3D) plot of the three properties (T_g_, CW, and CTE) for 135 novel PIs, along with screening results for high‐performance PIs. The right panel displays structural examples of 12 PIs with superior overall performance. (b–d) Predicted values of T_g_, CW, CTE, and SAScore for the 135 PIs. CTE, coefficient of thermal expansion; CW, cut‐off wavelength; PI, polyimide; T_g_, glass transition temperature.

Finally, we designed 135 novel PIs and obtained their T_g_, CW, and CTE through predictions using the three models. The study revealed that some PIs exhibited high T_g_, low CW, and low CTE. Twelve PIs were selected based on these properties, and their molecular structures are illustrated in Figure 6a. Synthetic accessibility score (SAscore) is a critical parameter for practical production, where an SAscore <6 indicates ease of synthesis.^[51]^ As shown in Figure 6b–d, all novel PIs had SAscore values below 7, with only six exceeding 6. The selected 12 PIs satisfied the balanced requirements for the three properties while remaining synthetically accessible. Predicted values for the three properties and SAscore are listed in Supporting Information S1: Table S9. Notably, certain PIs with singularly high performance may find applications in specialized fields; structural details, predicted properties, and SAscore for these PIs are provided in Supporting Information S1: Table S10. By integrating model predictions with SAscore, this approach enables structure selection based on desired PI properties while quantifying synthetic difficulty, thereby significantly reducing experimental costs, shortening development cycles, and improving research efficiency.

## 3 CONCLUSIONS

When synthesizing novel PIs, one cannot consider only a single property. To discover more promising PIs with better performance, we established predictive models for T_g_, CW and CTE using various ML algorithms. Among the models, the T_g_ and CW prediction models trained with CATB and the CTE prediction model trained with RF demonstrated the highest accuracy. The optimal predictive models for the three properties demonstrated high predictability and stability in both the test set and cross‐validation results. Through the SHAP method, interpretability analysis of the three optimal models revealed that the NumRotatableBonds, BCUT2D_LOGPLOW and BCUT2D_CHGLO descriptors are more important than others, exerting the most significant influence on the three properties, respectively. Using the three optimal models, we predicted the T_g_, CW and CTE of novel PI structures, verifying the accuracy and generalization capabilities of the models. Then, by combining the three models, we predicted multiple PIs, screened them, and synthesized those with superior comprehensive performance. Finally, 135 novel PIs were designed and their key properties were obtained without the need of experimental verification. Through screening, some PI candidates with higher overall performance and easy synthesis can be identified. The predictive models established in this study can help researchers quickly determine the T_g_, CW and CTE of PIs, thereby rapidly identifying candidates worthy of further development. Future work could incorporate molecular weight data or synthesis processes into ML models, as well as use ML to predict the dielectric and mechanical properties of PI materials, which will shorten development cycles, reduce experimental costs, and enhance the efficiency of new material research and development. This work advances data‐driven material development and provides new insights into the discovery of high‐performance polymers for the future.

## 4 METHODS

### 4.1 Dataset and feature extraction

Firstly, the repeating unit structures, T_g_, CW and CTE of PI materials, were collected from the PolyInfo database (http://polymer.nims.go.jp)^[52]^ and literature spanning from 2012 to 2024. Given that T_g_ data were obtained through various measurement methods, including thermomechanical analysis (TMA), DMA and DSC, differences in measurement methods could introduce discrepancies in T_g_ data; therefore, only T_g_ values measured by DSC were selected. Secondly, since the repeating units of PIs have unsaturated single bonds at both ends, hydrogen atoms were added to cap these ends, transforming the repeating units into complete molecular structures, which were then converted into SMILES expressions‐a linear notation system using ASCII strings to represent polymer structures.^[53]^ Finally, 210 molecular descriptors usable for ML were calculated using the open‐source cheminformatics library RDKit (https://www.rdkit.org) based on the SMILES expressions. Due to the risk of overfitting when using too many input descriptors, which can increase model complexity and reduce prediction accuracy, the descriptors were evaluated to identify those that significantly impact the model, thereby reducing computational time and improving predictive performance.[^38^, ^54^, ^55^] Initially, the descriptors were standardized, scaling the data to the range [0, 1] to eliminate dimensional differences between descriptors. Variance selection was then applied to calculate the variance of each descriptor, setting a threshold of 0.01 to select descriptors with variances greater than this threshold. Finally, key descriptors were further selected using the feature importance^[56]^ method.

### 4.2 ML model training

In this study, we utilize six ensemble learning algorithms—Random Forest (RF),^[57]^ Extremely Randomized Trees (ET),^[58]^ XGBoost (XGB),^[59]^ Gradient Boosting Regression (GBR),^[60]^ LightGBM (LGBM),^[61]^ and CATBoost (CATB)^[62]^—as well as a DNN^[63]^ deep learning algorithm to construct predictive models and investigate the impact of different algorithms on model accuracy. We divide the entire dataset into training and test sets, with specific allocations detailed in Supporting Information S1: Table S11. The test set is not involved in the model‐building process, and the error between experimental values and predicted values can reflect the predictive capability of the model to some extent. During the model training phase, we employ the GridSearchCV method to find the optimal hyperparameters for each ensemble learning approach, continuously adjusting parameters such as the number of hidden layers, the number of nodes, activation functions and loss functions in the DNN to ensure that the accuracy of the trained models is maximized for each algorithm. Finally, 10‐fold cross‐validation is used to test the stability of the models. Throughout this process, the following statistical parameters were used to evaluate the goodness of fit for each model: *R* ^2^, MAE and RMSE. The definitions of these statistical parameters are detailed in Supporting Information S1: Table S12.

### 4.3 Model interpretation and validation

We use SHAP to interpret the three optimal models individually, quantitatively explaining the relationship between descriptors and the predicted properties through the calculation of Shapley values. To verify the accuracy and generalization capability of the models, the optimal models were used to predict the properties of PIs reported in the literature from 2024. The structures of these PIs do not appear in the dataset and exhibit significant differences compared to the PIs within the dataset.

### 4.4 Screening and synthesis of PI candidates

By combining the three predictive models, we predicted the properties of PI within the T_g_ dataset and screen for PIs with T_g_ ≥ 340°C, CW ≤ 360 nm, and CTE ≤ 50 ppm/K, indicating superior comprehensive performance. Some PIs have only experimental data for T_g_, so we synthesized these in the laboratory and tested their T_g_, CW and CTE. Comparing the predicted values with the experimental values serves to validate the predictions of the models. Finally, 135 novel PIs were designed and their key properties were obtained without the need of experimental verification. Through screening, some candidates for PIs with overall higher performance and easy synthesis, as well as single high‐performance PIs can be identified.

## CONFLICT OF INTEREST STATEMENT

The authors declare no conflicts of interest.

## ETHICS STATEMENT

No animal or human experiments were involved in this study.

## Supporting information

Supporting Information S1

## Data Availability

The code for these three predictive models, the generated SMILES and descriptor data, and the 135 newly designed PIs are available on GitHub: https://github.com/wsstju/SMILES‐and‐descriptor.
