# Supplementary material for: Multifunctional polyimide performance prediction based on explainable machine learning
Source: Smart Mol. 2025 Oct 30;3(4):e70020. doi: 10.1002/smo2.70020 (PMC12755225; doi:10.1002/smo2.70020)
Supplement: Supplementary file 1 — Supporting Information S1 [file SMO2-3-e70020-s001.docx]

Supporting Information

**Multifunctional Polyimide Performance Prediction Based on Explainable Machine Learning**

*Suisui Wang**^+,[1]^, Tianyong Zhang^+,[1]^, Han Zhang^[2]^, Wenxuan Zhu^[1]^, Zixu Gu^[1]^, Xufeng Huang^[1]^, Hande Zhang^[1]^, Bin Li^*[1]^, Jianhua Zhang^*[2]^*

^+^ Suisui Wang and Tianyong Zhang contributed equally

[1] S. Wang, T. Zhang, W. Zhu, Z. Gu, X. Huang, H. Zhang, B. Li
School of Chemical Engineering and Technology
Tianjin University
Tianjin 300354, P. R. China
E-mail: libin@tju.edu.cn

[2] H. Zhang, J. Zhang
School of Microelectronics
Shanghai University
Shanghai 201800, P. R. China
E-mail: jhzhang@oa.shu.edu.cn

# Note S1 Supplementary detailed parameter settings

**Parameter setting for the CATB algorithm predicting T_g_**

best_param = {

'iterations': 1100,

'depth': 6,

'learning_rate': 0.09,

'loss_function': 'RMSE'

}

best_catb = CatBoostRegressor(**best_param)

best_catb.fit(X_train, y_train, verbose=False)

**Parameter setting for the CATB algorithm predicting CW**

best_param = {

'iterations': 900,

'depth': 4,

'learning_rate': 0.1,

'loss_function': 'RMSE'

}

best_catb = CatBoostRegressor(**best_param)

best_catb.fit(X_train, y_train, verbose=False)

**Parameter setting for the RF algorithm predicting CTE**

best_param = {

'n_estimators': 150,

'max_depth': 16,

'max_features': 8,

'min_samples_leaf': 1,

'min_samples_split': 1

}

best_rf = RandomForestRegressor(**best_param)

best_rf.fit(X_train, y_train)

# Note S2 The specific preparation method of PI materials

**Materials**

4,4''-Oxydiphthalicanhydride (ODPA), 4,4'-(9-fluorenylidene) dianiline and 4,4'-diaminodiphenyl ether (ODA) were purchased from Tianjin Sinensia Optode Technology Co., Ltd. Bicyclo[2.2.2]oct-7-ene-2,3,5,6-tetracarboxylic dianhydride (BOCA) was purchased from Bide Pharmatech Co., Ltd. Anhydrous N,N-dimethylacetamide (DMAc) and triethylamine were purchased from Energy Chemical. Acetic anhydride purchased from Lianlong Bohua (Tianjin) Pharmaceutical Chemical Co., Ltd.

**Synthesis of PI_1_ and PI_2_**

PI_1_: The Schlenk reaction flask was repeatedly evacuated and flushed with nitrogen three times to remove moisture and air. Then, 4,4'-(9-fluorenylidene) dianiline (1.74 g, 5.00 mmol) and 19.1 mL of DMAc solvent were added. After stirring until the diamine was completely dissolved, dianhydride ODPA (1.63 g, 5.10 mmol) was added, and the reaction mixture was stirred at 25 °C for 24 h to obtain a polyamic acid (PAA) solution with a solid content of 15 wt%. Triethylamine (1.0 g) and acetic anhydride (1.0 g) were added to the PAA solution as a catalyst and dehydrating agent, and the temperature was raised to 60 ℃ for 6 h to undergo chemical imidization, resulting in a viscous PI solution. This solution was then precipitated in a mixture of anhydrous methanol and water (1:1 ratio). After thorough washing with anhydrous methanol, the solution was placed in an oven at 100 °C for 24 h to dry, yielding white PI solids.

PI_2_: The Schlenk reaction flask was repeatedly evacuated and flushed with nitrogen three times to remove moisture and air. Then, ODA (1.00 g, 5.00 mmol) and 12.7 mL of DMAc solvent were added. After stirring until the diamine was completely dissolved, dianhydride BOCA (1.24 g, 5.10 mmol) was added, the mixture was then heated to 80 ℃ and maintained for 2 h, followed by the addition of five drops of isoquinoline. The temperature was increased to 180 ℃ and maintained for 12 h. After cooling to room temperature, the mixture was dropped into an anhydrous methanol solution for precipitation. The precipitate was repeatedly washed with anhydrous methanol until the filtrate was nearly colorless and clear. It was then dried in a vacuum oven at 100 ℃ for 24 h, yielding a light-yellow PI solid.

**Film fabrication**

Dissolve 0.5 g of PI solid in 5.0 mL of DMAc solvent and stir magnetically overnight. Using a pipette, precisely dispense 2.0 mL of the solution onto a 50 mm × 50 mm × 2 mm quartz plate for casting into a film. Heat the plate to 60 ℃ on a hot stage and maintain this temperature for 2 h; then increase the temperature to 80 ℃ and maintain for another 2 h. Place the plate in a vacuum drying oven at 100 ℃ for 12 h to completely remove the solvent and thoroughly dry the film. Finally, soak the film in deionized water to release it, resulting in a tough and flexible PI film.

# Supplementary Figures


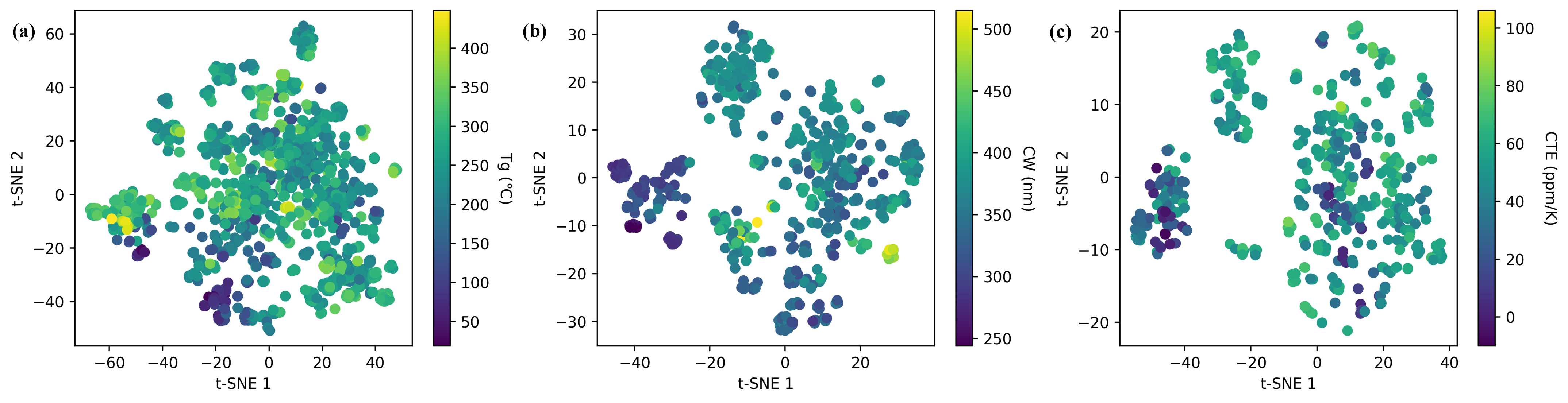


**Figure S1** 2D visualization based on Morgan molecular fingerprints using the t-SNE algorithm. (a) T_g_ dataset; (b) CW dataset; (c) CTE dataset.

**Figure S2** Results of the T_g_ prediction models trained by (a) RF; (b) ET; (c) XGB; (d) LGBM; (e) CATB; (f) DNN algorithms and prediction error (PE) distribution of training set and test set.

**Figure S3** Results of the CW prediction models trained by (a) RF; (b) ET; (c) GBR; (d) LGBM; (e) CATB; (f) DNN algorithms and prediction error (PE) distribution of training set and test set.

**Figure S4** Results of the CTE prediction models trained by (a) RF; (b) ET; (c) GBR; (d) XGB; (e) CATB; (f) DNN algorithms and prediction error (PE) distribution of training set and test set.


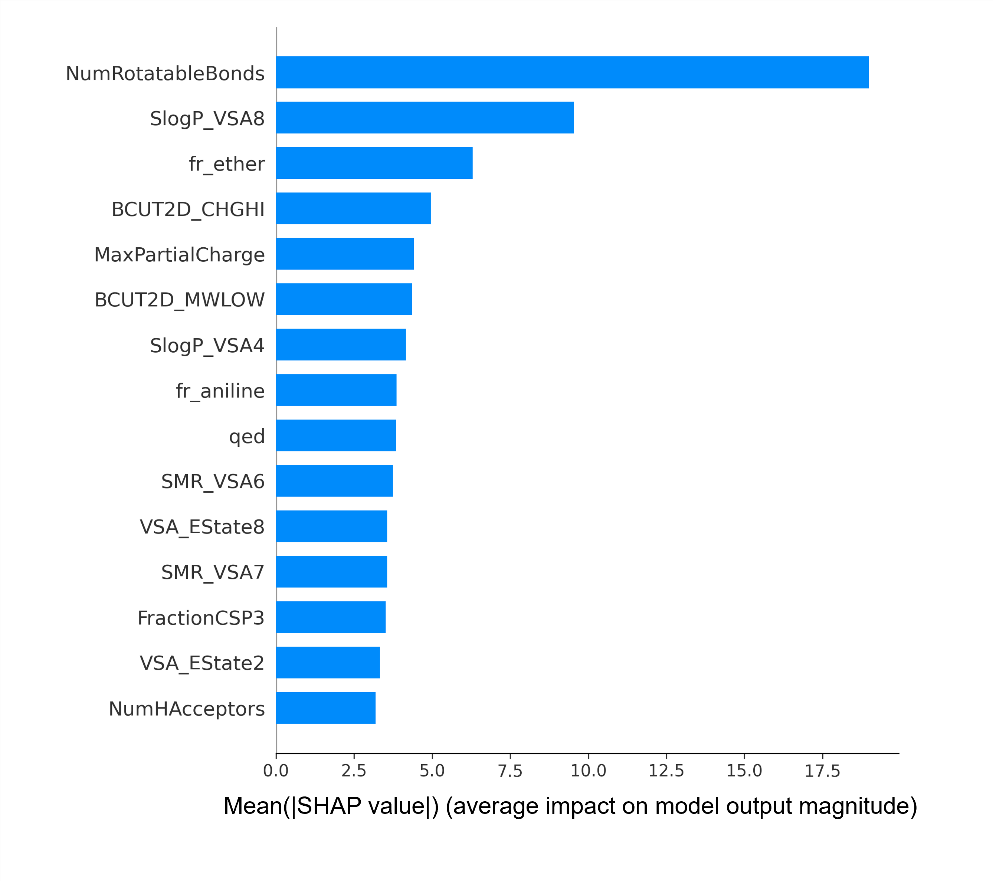


**Figure S5** The mean absolute SHAP value of each descriptor in the CATB algorithm for predicting T_g_.


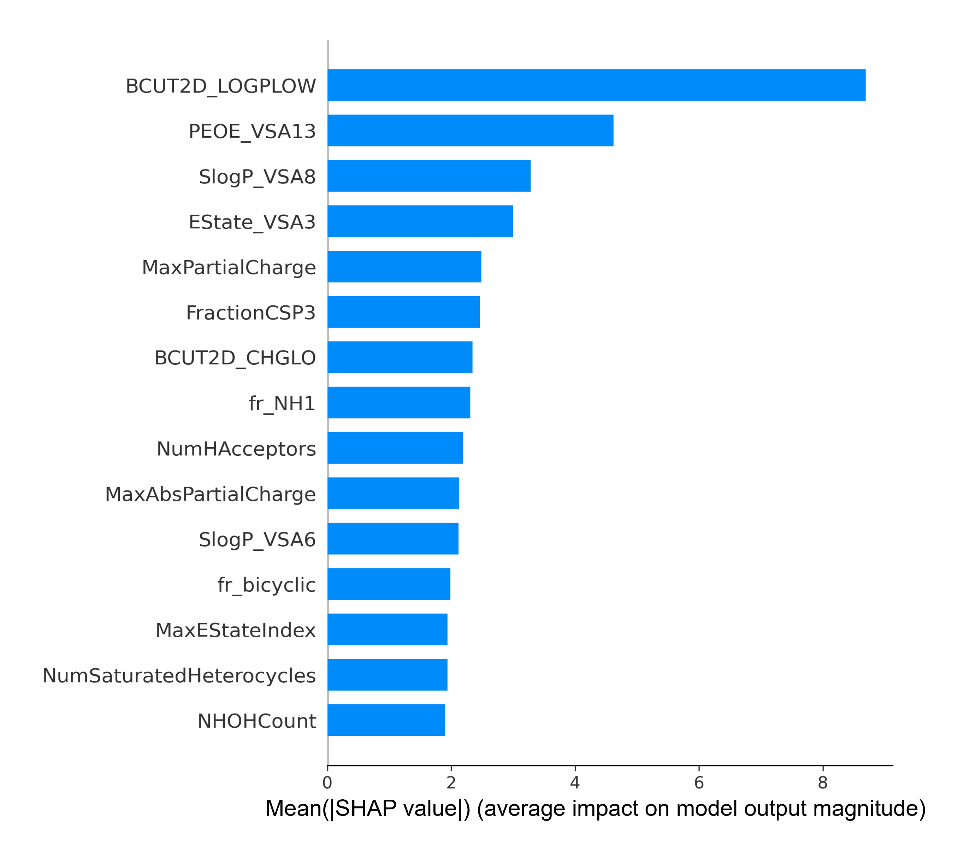


**Figure S6** The mean absolute SHAP value of each descriptor in the CATB algorithm for predicting CW.


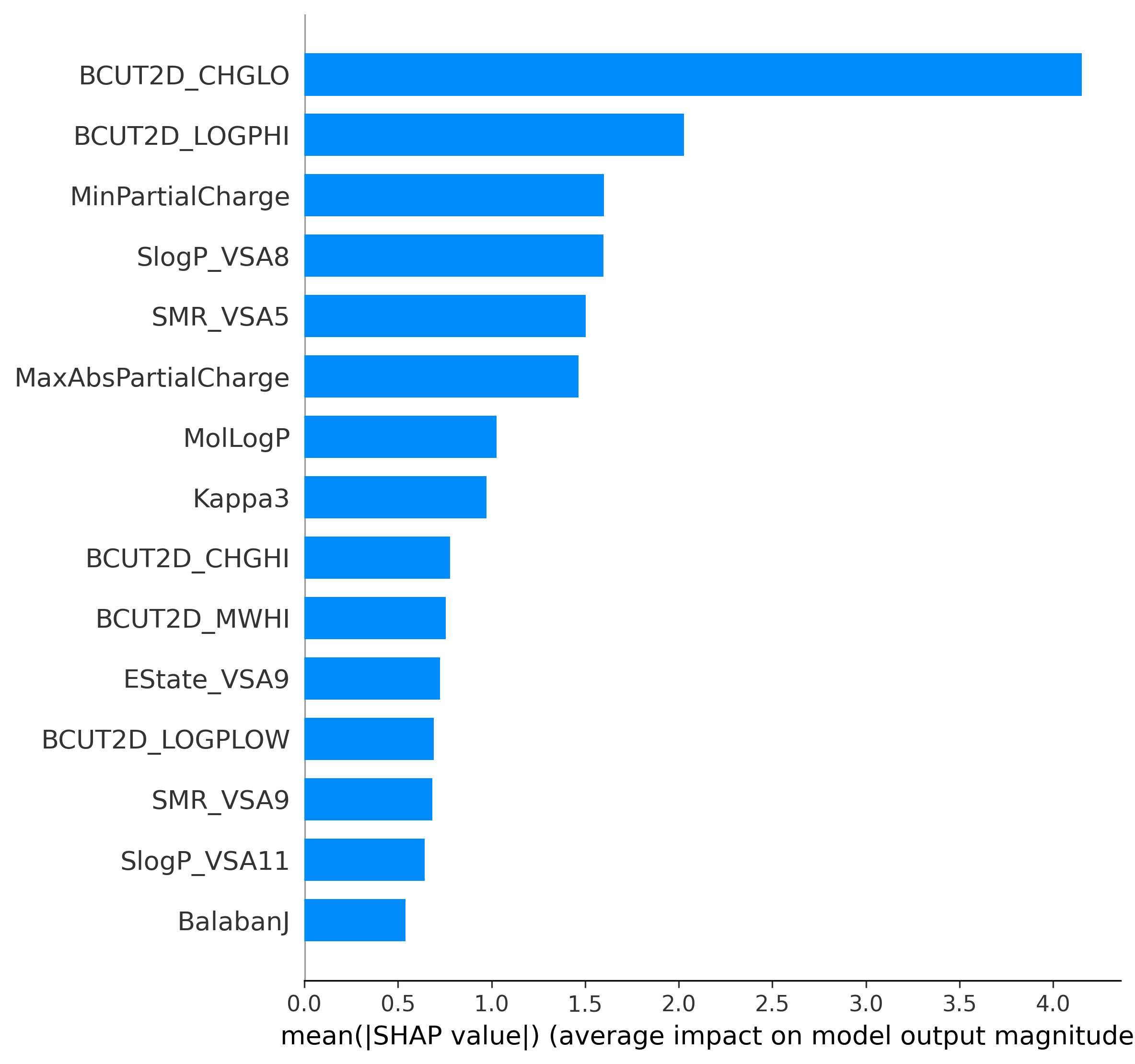


**Figure S7** The mean absolute SHAP value of each descriptor in the RF algorithm for predicting CTE.


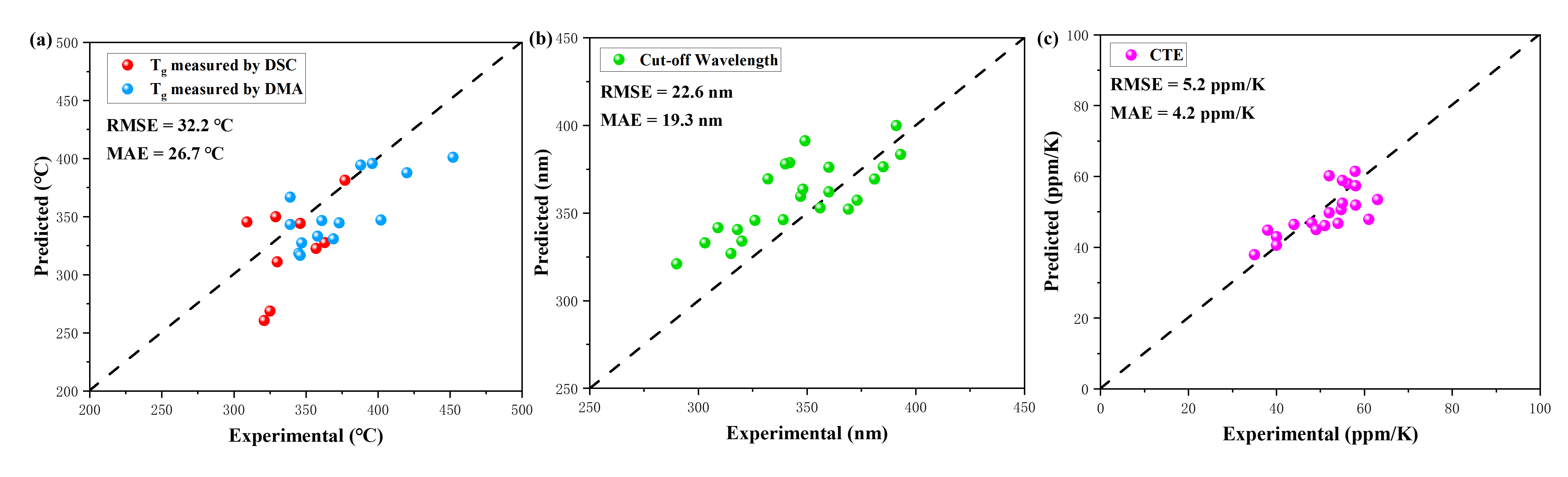


**Figure S8** Comparison between experimental values of the new PI and the values predicted by the model: (a) T_g_, (b) CW, (c) CTE.


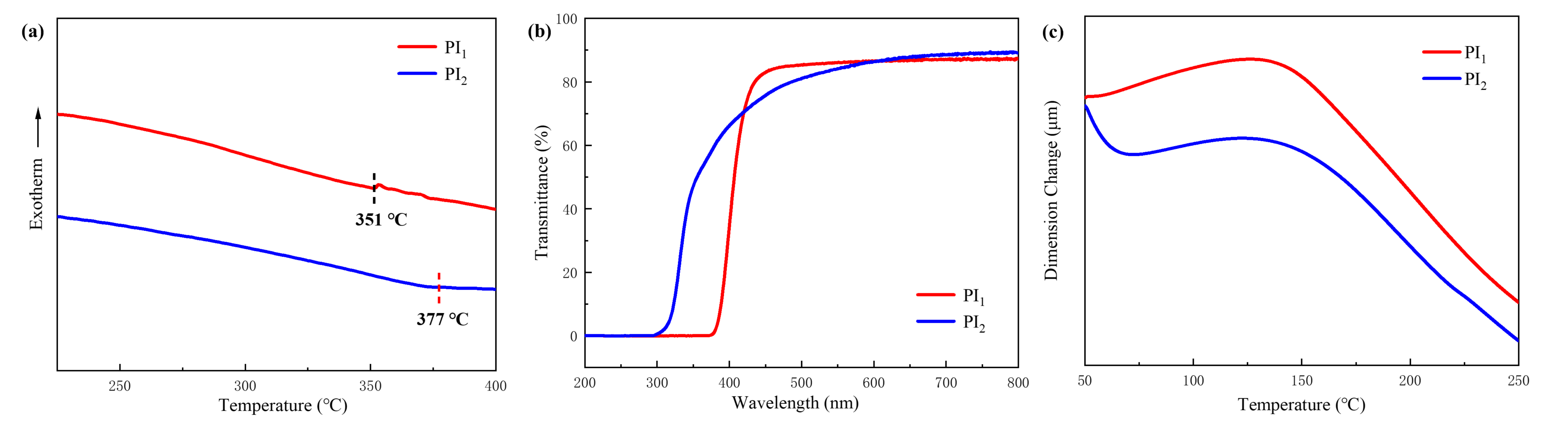


**Figure S9** Experimental values of the three properties of PI_1_ and PI_2_: (a) T_g_, (b) CW, (c) CTE.


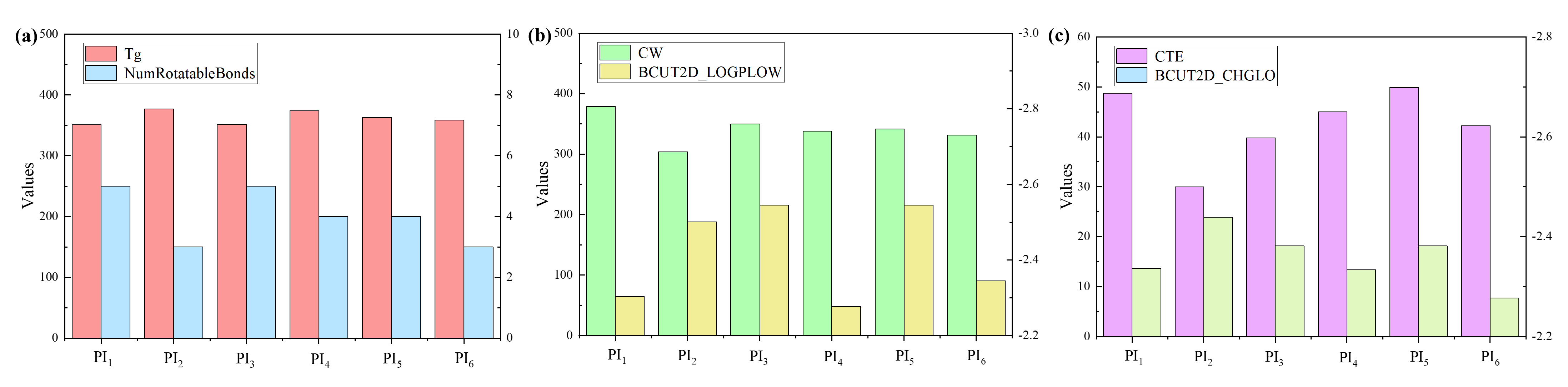


**Figure S10** Important descriptors for PI structure calculation with experimental performance characterization values: (a) NumRotatableBonds descriptor and T_g_, (b) BCUT2D_LOGPLOW descriptor and CW, (c) BCUT2D_CHGLO descriptor and CTE.

# Supplementary Tables

**Table S1** Number of selected descriptors used by different ML methods for the three properties

| Property | ML algorithms | Number of selected descriptors |
| --- | --- | --- |
| T_g_ | RF | 26 |
|  | ET | 30 |
|  | XGB | 18 |
|  | LGBM | 34 |
|  | CATB | 26 |
|  | DNN | 131 |
| CW | RF | 16 |
|  | ET | 24 |
|  | GBR | 22 |
|  | LGBM | 30 |
|  | CATB | 27 |
|  | DNN | 140 |
| CTE | RF | 18 |
|  | ET | 25 |
|  | GBR | 17 |
|  | XGB | 22 |
|  | CATB | 23 |
|  | DNN | 144 |

**Table S2** Comparison of T_g_ prediction models using different algorithms

| Model | Training dataset | | | Test dataset | | | Cross-validation results ^a^ | | |
| --- | --- | --- | --- | --- | --- | --- | --- | --- | --- |
|  | R^2^ | MAE (℃) | RMSE (℃) | R^2^ | MAE (℃) | RMSE (℃) | R^2^ | MAE (℃) | RMSE (℃) |
| RF | 0.966 | 9.3 | 12.2 | 0.825 | 22.1 | 29.2 | 0.811±0.026 | 21.6±1.1 | 29.0±1.9 |
| ET | 0.988 | 4.1 | 7.2 | 0.851 | 19.2 | 27.0 | 0.833±0.023 | 19.5±0.64 | 27.3±1.1 |
| XGB | 0.965 | 9.0 | 12.5 | 0.816 | 21.7 | 29.9 | 0.803±0.015 | 21.4±0.94 | 29.8±1.5 |
| LGBM | 0.996 | 3.1 | 4.4 | 0.834 | 20.8 | 28.4 | 0.827±0.015 | 20.0±0.81 | 27.8±1.2 |
| CATB | 0.995 | 3.5 | 4.9 | 0.865 | 18.1 | 25.6 | 0.850±0.020 | 18.3±0.74 | 25.9±1.4 |
| DNN | 0.944 | 10.6 | 15.8 | 0.842 | 19.3 | 27.7 | 0.671±0.104 | 32.7±6.5 | 41.0±7.7 |

^a^ The error is the standard deviation of the cross-validation results

**Table S3** Comparison of CW prediction models using different algorithms

| Model | Training dataset | | | | Test dataset | | | Cross-validation results ^a^ | | |
| --- | --- | --- | --- | --- | --- | --- | --- | --- | --- | --- |
|  | R^2^ | MAE (nm) | | RMSE (nm) | R^2^ | MAE (nm) | RMSE (nm) | R^2^ | MAE (nm) | RMSE (nm) |
| RF | 0.967 | | 5.4 | 7.4 | 0.844 | 12.3 | 16.7 | 0.793±0.081 | 13.2±1.8 | 18.2±2.6 |
| ET | 0.993 | | 1.7 | 3.5 | 0.868 | 11.5 | 15.4 | 0.805±0.072 | 12.9±1.5 | 17.3±2.5 |
| GBR | 0.979 | | 4.3 | 6.0 | 0.839 | 12.1 | 16.9 | 0.816±0.067 | 12.7±1.6 | 17.0±2.2 |
| LGBM | 0.996 | | 1.6 | 2.6 | 0.822 | 12.7 | 17.8 | 0.778±0.078 | 13.7±1.4 | 18.7±2.1 |
| CATB | 0.984 | | 3.7 | 5.1 | 0.874 | 10.6 | 15.0 | 0.830±0.077 | 11.8±1.7 | 16.2±2.6 |
| DNN | 0.954 | | 6.0 | 8.7 | 0.867 | 11.2 | 15.4 | 0.768±0.059 | 14.9±1.9 | 18.5±2.5 |

^a^ The error is the standard deviation of the cross-validation results

**Table S4** Comparison of CTE prediction models using different algorithms

| Model | Training dataset | | | | Test dataset | | | Cross-validation results ^a^ | | |
| --- | --- | --- | --- | --- | --- | --- | --- | --- | --- | --- |
|  | R^2^ | MAE (ppm/K) | | RMSE (ppm/K) | R^2^ | MAE (ppm/K) | RMSE (ppm/K) | R^2^ | MAE (ppm/K) | RMSE (ppm/K) |
| RF | 0.962 | | 2.6 | 3.4 | 0.855 | 6.6 | 8.3 | 0.767±0.083 | 6.5±0.5 | 8.4±0.8 |
| ET | 0.997 | | 0.4 | 1.0 | 0.830 | 6.8 | 9.0 | 0.740±0.116 | 6.5±0.8 | 8.8±0.8 |
| GBR | 0.997 | | 0.3 | 0.9 | 0.847 | 6.3 | 8.5 | 0.750±0.110 | 6.5±0.8 | 8.6±0.8 |
| XGB | 0.981 | | 1.7 | 2.4 | 0.846 | 6.4 | 8.5 | 0.758±0.098 | 6.3±0.7 | 8.5±1.0 |
| CATB | 0.997 | | 0.3 | 0.9 | 0.834 | 6.5 | 8.9 | 0.733±0.115 | 6.8±0.8 | 8.9±0.8 |
| DNN | 0.864 | | 4.6 | 6.4 | 0.819 | 7.0 | 9.3 | 0.733±0.106 | 7.2±0.7 | 9.7±1.0 |

^a^ The error is the standard deviation of the cross-validation results

**Table S5** List of descriptors used by the CATB algorithm for predicting T_g_

| Descriptor name | Description |
| --- | --- |
| qed | Calculate the weighted sum of ADS mapped properties |
| MaxPartialCharge | Maximum partial charge. A partial charge is a non-integer charge value when measured in elementary charge units |
| MaxAbsPartialCharge | Maximum absolute partial charge. A partial charge is a non-integer charge value when measured in elementary charge units |
| BCUT2D_MWLOW | Implements BCUT descriptors from J. Chem. Inf. Comput. Sci., 1999 (39), No. 1 |
| BCUT2D_CHGHI | Implements BCUT descriptors from J. Chem. Inf. Comput. Sci., 1999 (39), No. 1 |
| BCUT2D_MRHI | Implements BCUT descriptors from J. Chem. Inf. Comput. Sci., 1999 (39), No. 1 |
| BertzCT | A topological index meant to quantify "complexity" of molecules |
| PEOE_VSA6 | MOE charge VSA descriptor 6 (-0.10 <= x < -0.05) |
| PEOE_VSA8 | MOE charge VSA descriptor 8 ( 0.00 <= x < 0.05) |
| PEOE_VSA9 | MOE charge VSA descriptor 9 ( 0.05 <= x < 0.10) |
| SMR_VSA6 | MOE MR VSA Descriptor 6 ( 2.75 <= x < 3.05) |
| SMR_VSA7 | MOE MR VSA Descriptor 7 ( 3.05 <= x < 3.63) |
| SlogP_VSA1 | MOE logP VSA Descriptor 1 (-inf < x < -0.40) |
| SlogP_VSA11 | MOE logP VSA Descriptor 11 ( 0.50 <= x < 0.60) |
| SlogP_VSA4 | MOE logP VSA Descriptor 4 ( 0.00 <= x < 0.10) |
| SlogP_VSA8 | MOE logP VSA Descriptor 8 ( 0.25 <= x < 0.30) |
| VSA_EState2 | VSA EState Descriptor 2 ( 4.78 <= x < 5.00) |
| VSA_EState8 | VSA EState Descriptor 8 ( 6.45 <= x < 7.00) |
| FractionCSP3 | CalcFractionCSP3( (Mol)mol) -> float : returns the fraction of C atoms that are SP3 hybridized |
| NumAromaticRings | CalcNumAromaticRings( (Mol)mol) -> int : returns the number of aromatic rings for a molecule |
| NumHAcceptors | Number of hydrogen bond acceptors |
| NumRotatableBonds | Number of rotatable bonds |
| RingCount | Number of rings in the molecule |
| fr_aniline | Number of anilines |
| fr_bicyclic | Bicyclic |
| fr_ether | Number of ether oxygens (including phenoxy) |

**Table S6** List of descriptors used by the CATB algorithm for predicting CW

| Descriptor name | Description |
| --- | --- |
| MaxEStateIndex | Maximum EState index |
| SPS | Calculating the spatial score of molecules to measure their steric structure |
| MaxPartialCharge | Maximum partial charge. A partial charge is a non-integer charge value when measured in elementary charge units |
| MinPartialCharge | Minimum partial charge. A partial charge is a non-integer charge value when measured in elementary charge units |
| MaxAbsPartialCharge | Maximum absolute partial charge. A partial charge is a non-integer charge value when measured in elementary charge units |
| MinAbsPartialCharge | Minimum absolute partial charge. A partial charge is a non-integer charge value when measured in elementary charge units |
| BCUT2D_MWLOW | Implements BCUT descriptors from J. Chem. Inf. Comput. Sci., 1999 (39), No. 1 |
| BCUT2D_CHGLO | Implements BCUT descriptors from J. Chem. Inf. Comput. Sci., 1999 (39), No. 1 |
| BCUT2D_LOGPHI | Implements BCUT descriptors from J. Chem. Inf. Comput. Sci., 1999 (39), No. 1 |
| BCUT2D_LOGPLOW | Implements BCUT descriptors from J. Chem. Inf. Comput. Sci., 1999 (39), No. 1 |
| PEOE_VSA12 | MOE charge VSA descriptor 12 ( 0.20 <= x < 0.25) |
| PEOE_VSA13 | MOE charge VSA descriptor 13 ( 0.25 <= x < 0.30) |
| SMR_VSA4 | MOE MR VSA Descriptor 4 ( 2.24 <= x < 2.45) |
| SMR_VSA5 | MOE MR VSA Descriptor 5 ( 2.45 <= x < 2.75) |
| SlogP_VSA4 | MOE logP VSA Descriptor 4 ( 0.00 <= x < 0.10) |
| SlogP_VSA6 | MOE logP VSA Descriptor 6 ( 0.15 <= x < 0.20) |
| SlogP_VSA8 | MOE logP VSA Descriptor 8 ( 0.25 <= x < 0.30) |
| EState_VSA3 | EState VSA Descriptor 3 ( 0.29 <= x < 0.72) |
| VSA_EState8 | VSA EState Descriptor 8 ( 6.45 <= x < 7.00) |
| FractionCSP3 | CalcFractionCSP3( (Mol)mol) -> float : returns the fraction of C atoms that are SP3 hybridized |
| NHOHCount | Number of NHs or OHs |
| NumHAcceptors | Number of hydrogen bond acceptors |
| NumSaturatedHeterocycles | CalcNumSaturatedHeterocycles( (Mol)mol) -> int : returns the number of saturated heterocycles for a molecule |
| NumSaturatedRings | CalcNumSaturatedRings( (Mol)mol) -> int : returns the number of saturated rings for a molecule |
| fr_NH1 | Number of secondary amines |
| fr_bicyclic | Bicyclic |
| fr_para_hydroxylation | Number of para-hydroxylation sites |

**Table S7** List of descriptors used by the RF algorithm for predicting CTE

| Descriptor name | Description |
| --- | --- |
| MinAbsEStateIndex | The absolute value of minimum E-state of the molecule |
| SPS | Calculating the spatial score of molecules to measure their steric structure |
| MinPartialCharge | Minimum partial charge. A partial charge is a non-integer charge value when measured in elementary charge units |
| MaxAbsPartialCharge | Maximum absolute Partial Charge. A partial charge is a non-integer charge value when measured in elementary charge units |
| BCUT2D_MWHI | Implements BCUT descriptors from J. Chem. Inf. Comput. Sci., 1999 (39), No. 1 |
| BCUT2D_CHGHI | Implements BCUT descriptors from J. Chem. Inf. Comput. Sci., 1999 (39), No. 1 |
| BCUT2D_CHGLO | Implements BCUT descriptors from J. Chem. Inf. Comput. Sci., 1999 (39), No. 1 |
| BCUT2D_LOGPHI | Implements BCUT descriptors from J. Chem. Inf. Comput. Sci., 1999 (39), No. 1 |
| BCUT2D_MRHI | Implements BCUT descriptors from J. Chem. Inf. Comput. Sci., 1999 (39), No. 1 |
| BCUT2D_MRLOW | Implements BCUT descriptors from J. Chem. Inf. Comput. Sci., 1999 (39), No. 1 |
| BalabanJ | Calculate Balaban's J value for a molecule |
| Kappa3 | Hall-Kier Kappa3 value |
| SMR_VSA5 | MOE MR VSA Descriptor 5 ( 2.45 <= x < 2.75) |
| SMR_VSA9 | MOE MR VSA Descriptor 9 ( 3.80 <= x < 4.00) |
| SlogP_VSA11 | MOE logP VSA Descriptor 11 ( 0.50 <= x < 0.60) |
| SlogP_VSA8 | MOE logP VSA Descriptor 8 ( 0.25 <= x < 0.30) |
| EState_VSA9 | EState VSA Descriptor 9 ( 4.69 <= x < 9.17) |
| MolLogP | Wildman-Crippen LogP value |

**Table S8** Comparison of experimental and predicted values for the three properties

| Structure of the repeating unit | T_g_ (℃) | | | CW (nm) | | CTE (ppm/K) | | Reference |
| --- | --- | --- | --- | --- | --- | --- | --- | --- |
|  | Experimental | Measurement method | Predicted | Experimental | Predicted | Experimental | Predicted |  |
| 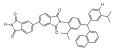 | 330 | DSC | 311 | 360 | 362 | 54.7 | 50.6 | ^[1]^ |
| 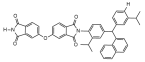 | 321 | DSC | 261 | 339 | 346 | 57.9 | 61.5 | ^[1]^ |
| 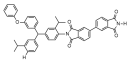 | 325 | DSC | 269 | 347 | 359 | 56.3 | 57.9 | ^[1]^ |
| 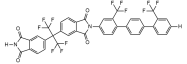 | 339 | DMA | 343 | 326 | 346 | 48 | 46.9 | ^[2]^ |
| 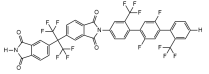 | 345 | DMA | 319 | 320 | 334 | 51 | 46.1 | ^[2]^ |
| 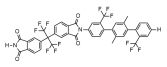 | 361 | DMA | 347 | 309 | 342 | 49 | 45.0 | ^[2]^ |
| 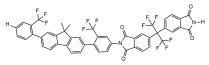 | 339 | DMA | 367 | 356 | 353 | 44 | 46.4 | ^[2]^ |
| 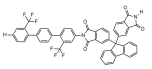 | 388 | DMA | 395 | 349 | 391 | 40 | 43.0 | ^[2]^ |
| 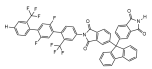 | 396 | DMA | 396 | 342 | 379 | 40 | 40.6 | ^[2]^ |
| 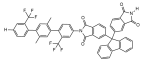 | 420 | DMA | 388 | 340 | 378 | 38 | 44.8 | ^[2]^ |
| 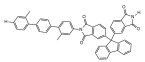 | 452 | DMA | 401 | 391 | 400 | 35 | 37.9 | ^[2]^ |
| Structure of the repeating unit | T_g_ (℃) | | | CW (nm) | | CTE (ppm/K) | | Reference |
|  | Experimental | Measurement method | Predicted | Experimental | Predicted | Experimental | Predicted |  |
| 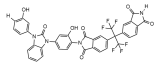 | 402 | DMA | 347 | 360 | 376 | 58 | 51.9 | ^[3]^ |
| 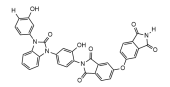 | 373 | DMA | 345 | 393 | 383 | 52 | 49.8 | ^[3]^ |
| 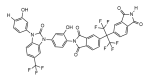 | 369 | DMA | 331 | 332 | 369 | 63 | 53.5 | ^[3]^ |
| 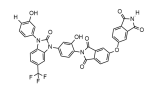 | 347 | DMA | 328 | 381 | 370 | 55 | 52.5 | ^[3]^ |
| 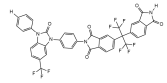 | 358 | DMA | 333 | 373 | 357 | 61 | 47.9 | ^[3]^ |
| 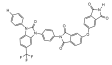 | 346 | DMA | 317 | 385 | 376 | 54 | 46.7 | ^[3]^ |
| 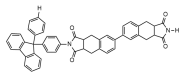 | 377 | DSC | 381 | 315 | 327 | - | - | ^[4]^ |
| 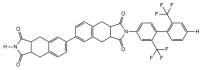 | 309 | DSC | 346 | 303 | 333 | - | - | ^[4]^ |
| 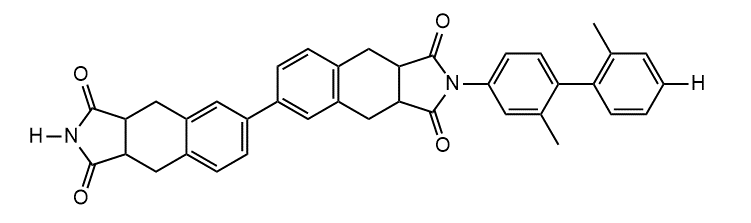 | 329 | DSC | 350 | 290 | 321 | - | - | ^[4]^ |
| 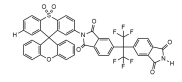 | 346 | DSC | 344 | 318 | 340 | 55 | 58.9 | ^[5]^ |
| 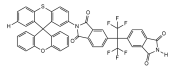 | 357 | DSC | 323 | 369 | 352 | 58 | 57.4 | ^[5]^ |
| 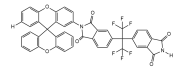 | 363 | DSC | 328 | 348 | 364 | 52 | 60.2 | ^[5]^ |

**Table S9** Predicted values of T_g_, CW, CTE for 12 novel PIs and their SAscore

| PI | Predicted value of T_g_ (℃) | Predicted value of CW (nm) | Predicted value of CTE (ppm/K) | SAscore |
| --- | --- | --- | --- | --- |
| PI-1 | 352.9 | 342.5 | 44.0 | 4.86 |
| PI-2 | 328.0 | 312.5 | 41.6 | 5.83 |
| PI-3 | 340.7 | 305.4 | 43.7 | 4.83 |
| PI-4 | 320.8 | 326.5 | 46.9 | 4.96 |
| PI-5 | 330.3 | 353.5 | 33.4 | 3.70 |
| PI-6 | 367.9 | 338.9 | 14.2 | 3.80 |
| PI-7 | 331.5 | 299.8 | 40.9 | 5.90 |
| PI-8 | 328.1 | 349.7 | 41.3 | 4.51 |
| PI-9 | 342.0 | 343.9 | 44.2 | 5.32 |
| PI-10 | 329.6 | 296.5 | 41.3 | 4.91 |
| PI-11 | 347.0 | 321.2 | 43.1 | 3.85 |
| PI-12 | 332.0 | 316.5 | 46.7 | 4.97 |

**Table S10** Structure and T_g_, CW, and CTE predicted values of a single high-performance new type of PIs and their SAscore

| Single high performance | Structure of the repeating unit | Predicted value of T_g_ (℃) | Predicted value of CW (nm) | Predicted value of CTE (ppm/K) | SAScore |
| --- | --- | --- | --- | --- | --- |
| T_g_ | 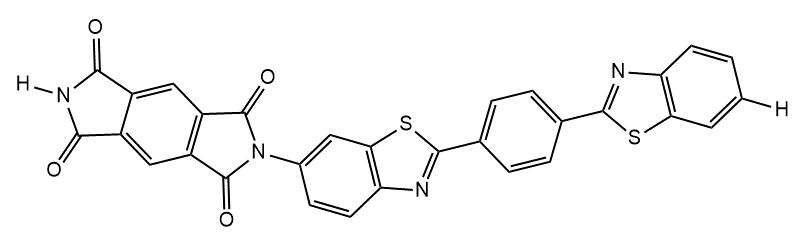 | **385** | 457 | 18.3 | 3.54 |
|  | 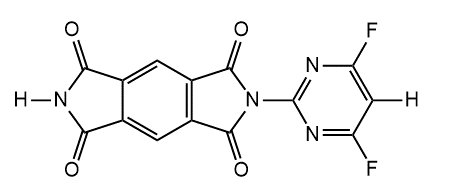 | **380** | 382 | 26 | 4.00 |
|  | 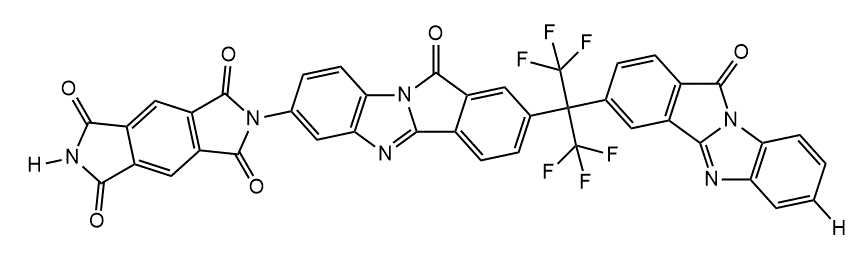 | **374** | 377 | 47.3 | 4.27 |
| CW | 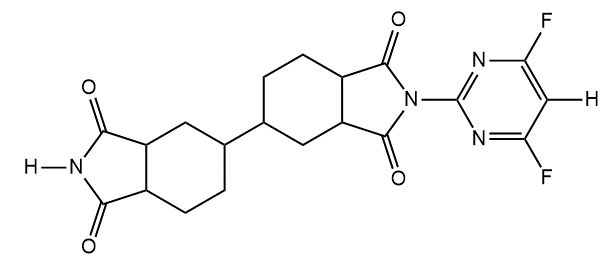 | 272 | **285** | 52.0 | 5.22 |
|  | 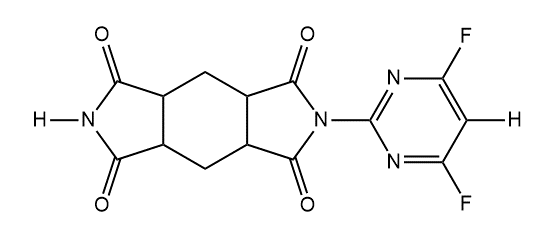 | 283 | **286** | 42.3 | 5.12 |
|  | 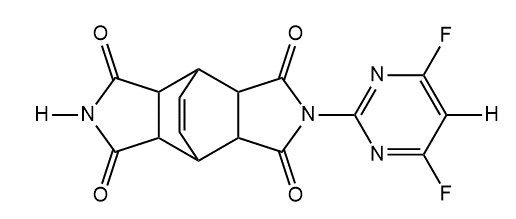 | 286 | **290** | 40.1 | 6.09 |
| CTE | 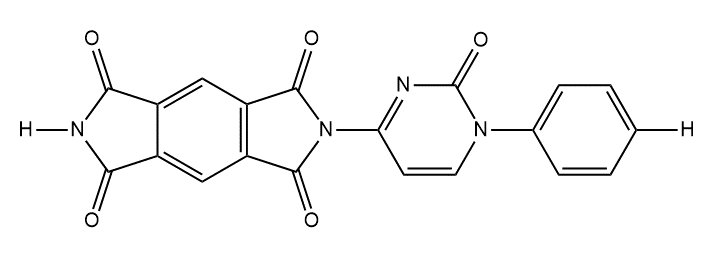 | 332 | 365 | **8.3** | 3.73 |
|  | 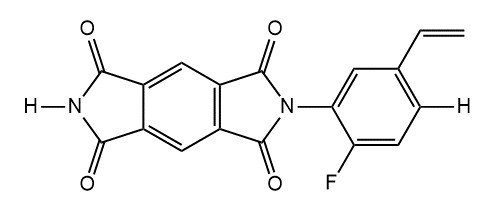 | 368 | 339 | **14.2** | 3.80 |
|  | 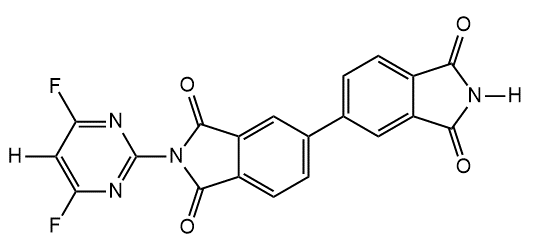 | 349 | 384 | **18.1** | 3.62 |

**Table S11** Division of training and test sets for the three properties

| Property | Dataset (Training set/test set) | Source | Unit |
| --- | --- | --- | --- |
| T_g_ | 1674 (1339/335) | Experimental | ℃ |
| CW | 623 (498/125) | Experimental | nm |
| CTE | 512 (409/103) | Experimental | ppm/K |

**Table S12** Statistical parameters mentioned in the article

| Parameters | Definition |
| --- | --- |
| Correlation coefficient (R) | $R=\sqrt{1-\frac{\sum_{i=1}^{n} {(T_{g_{i, exp}}-T_{g_{i,pred}})}^{2}}{\sum_{i=1}^{n} \left( T_{g_{i,exp}}-\bar{T}_{g_{exp}} \right)^{2}}}$ |
| Determination coefficient (R^2^) | $R^{2}=1-\frac{\sum_{i=1}^{n} {(T_{g_{i, exp}}-T_{g_{i,pred}})}^{2}}{\sum_{i=1}^{n} \left( T_{g_{i,exp}}-\bar{T}_{g_{exp}} \right)^{2}}$ |
| Mean absolute error (MAE) | $MAE=\frac{1}{n}\sum_{i=1}^{n} \left\vert T_{g_{i, exp}}-T_{g_{i,pred}} \right\vert$ |
| Root mean squared error (RMSE) | $RMSE=\sqrt{\frac{1}{n}\sum_{i=1}^{n} {(T_{g_{i, exp}}-T_{g_{i,pred}})}^{2}}$ |

Note: n is the number of PI, $T_{g_{i, exp}}$ is the experimental value of the i-th PI, $T_{g_{i,pred}}$ is the predicted value of the i-th PI, $\bar{T}_{g_{exp}}$ is the mean of all PI experimental values.

**References**

[1] Y. K. She, S. X. Wang, Q. Liao, M. J. Lin, *J. Polym. Sci.* **2023**, *62*, 1061-1073.

[2] Y. Fang, X. He, J. C. Kang, L. Wang, T. M. Ding, X. Lu, S. Y. Zhang, Q. Lu, *European Polymer Journal* **2024**, *202*.

[3] H. Zhu, Y. Su, J. Li, Y. Ding, M. Li, W. Li, *ACS Appl. Polym. Mater.* **2024**, *6*, 6130-6139.

[4] D. Zhou, L. L. Kong, S. M. Liu, Y. C. Yuan, Y. G. Ma, M. J. Huang, Y. Q. Mo, D. H. Qin, J. Q. Zhao, *Journal of Applied Polymer Science* **2023**, *141*.

[5] Z. Ning, Y. Yan, J. Wang, C. Zhao, L. Chen, L. Zong, Y. Xiao, *ACS Appl. Polym. Mater.* **2024**, *6*, 5434-5442.
